# Status of the management of hypertension and diabetes in rural Kenya: a multi-institutional joint intervention of cross-sectional surveys

**DOI:** 10.11604/pamj.2022.43.65.31347

**Published:** 2022-10-07

**Authors:** Ana Morales Ortiz, Robbinson Thindiu, Victor Lopez-Lopez, Maria Jose Gonzalez-Soriano, Herminia Pascual-Saura, Victorio Torres-Feced, Winnie Kanyi, Joseph Mbai, Maria Dolores Hernandez-Palazon, Miguel Gonzalez, Quiteria Hernández, José Gil Martinez, José M Rodriguez

**Affiliations:** 1 Department of Neurology, Virgen de la Arrixaca University Hospital, Murcia, IMIB-Arrixaca, Spain,; 2 Kigumo Sub County Hospital, Kigumo, Kenya,; 3 Department of Surgery, Virgen de la Arrixaca University Hospital Murcia, IMIB-Arrixaca, Spain,; 4 Department of Nephrology, Virgen de la Arrixaca University Hospital Murcia, IMIB-Arrixaca, Spain,; 5 Department of Endocrinology, Morales Meseguer University Hospital, Murcia, IMIB-Arrixaca, Spain,; 6 Department of Health Murang'a County, Muranga, Kenya,; 7 CEC Health & Sanitation Peoples Government of Murang'a County, Murang'a, Kenya,; 8 Department of Surgery, Reina Sofía University Hospital, Murcia, Spain

**Keywords:** Hypertension, diabetes, non-communicable diseases, sub-Saharan Africa

## Abstract

**Introduction:**

the aim of this study was to analyze the status of the management of vascular risk factors (hypertension and diabetes) at hospital level (3, 4 and 5) in Murang'a County (Kenya) health system.

**Methods:**

between July and December 2018 we performed a joint intervention about the strategies for improving care management of hypertension and diabetic patients in Murang'a (Kenya). A survey based on the recommendations from WHO about management of diabetes and hypertension was completed for 9 health-care centers. The survey made use of a semi-structured questionnaire, while the units of analysis for the survey were households.

**Results:**

the number of patients recorded at medical registers with diabetes and hypertension registered in public hospitals in Murang'a County were 6628 (0.45%) and y 6694 (0.45%), respectively. In the surveyed health-care centers, no hospital use electrogram and only one had troponin test. No hospital stocked Isosorbide dinitrate and Glicerine trinitate to prevent chest pain in patients with a heart condition. Only 3 of the clinics performed visual acuity examination and no facility did neurologic examination for neuropathy complications. No public hospital had HbA1 and did microalbuminuria test available.

**Conclusion:**

it is necessary to improve to establish screening methods, diagnosis, treatment and follow-up of patients with hypertension and diabetes in Murang'a County at the various levels of health care.

## Introduction

The Global Burden of Disease Study reports that non-communicable diseases (NCDs) account for three-quarters of all annual worldwide deaths in 2019 [1]. The main types of NCDs are cardiovascular diseases (like heart attacks and stroke), cancers, chronic respiratory diseases (such as chronic obstructive pulmonary disease and asthma) and diabetes [2]. According to World Health Organization (WHO) [3], ischemic heart disease alone is responsible for 16% of all deaths in the world, accounting for 8.9 million deaths in 2019 with an increase from 2 million deaths in the year 2000. It shows the largest increase in deaths for any other disease [4]. Ischemic heart disease and stroke occupy the positions 3 and 4 in low- and middle-income countries. Due to increasing life expectancy, rapid demographic transition and additional risk introduced by human immunodeficiency virus (HIV), WHO estimates that the African region will experience a large increase in the incidence of and mortality from NCD over the next decade [5]. Half of CVD (chronic vascular disease) deaths in sub-Saharan Africa occur among people aged between 30 and 69, which is 10 years earlier than in North America and Europe.

Therefore, it is very important that vascular risk factors (hypertension and diabetes) are well managed in these countries. WHO considers that these diseases must be controlled at the primary health care level and worked in initiatives such as STEPS (STEPwise approach to Surveillance) [6] and PEN (Package of essential non-communicable disease intervention) [7]. These tools are designed to monitor and manage these diseases in low-resource settings and provide evidence-based clinical guidelines to improve access and quality of NCD services provided in primary health care and hospital service settings. Kenya's population grew from 37.1 million in 2009 to about 47.5 million in 2019; it shows a growth of approximately 1 million people per year [8]. The expectation of life at birth (Life expectancy - LE) in Kenya had improved from a low of 45.2 years in the 1990s to an estimated 66,34 years by 2018 [9] and infant mortality rate in the year 2019 in Kenya is 31.8/1000 live births [10]. In recent years the profile of diseases is changing in Kenya with increased control of infectious diseases. People are not only living longer, but the number of NCD is increasing.

It is estimated that 25% of hospital admissions are due to CVD and 13% of autopsies revealed CVDs as cause of death, representing the second-highest cause of death after infectious/maternal/perinatal causes. In the recently published guidelines of cardiovascular management (Kenya national guidelines for cardiovascular diseases' management) [11], it is indicated that it is a priority to know what is the situation regarding the real number of hypertensive and diabetic patients in each county and how these diseases and their complications are being managed in Kenyan health system. The aim of this study was to update the status of the management of vascular risk factors (hypertension and diabetes) at hospital level (4 and 5) in Murang'a County (Kenya).

## Methods

**Figure 1:**
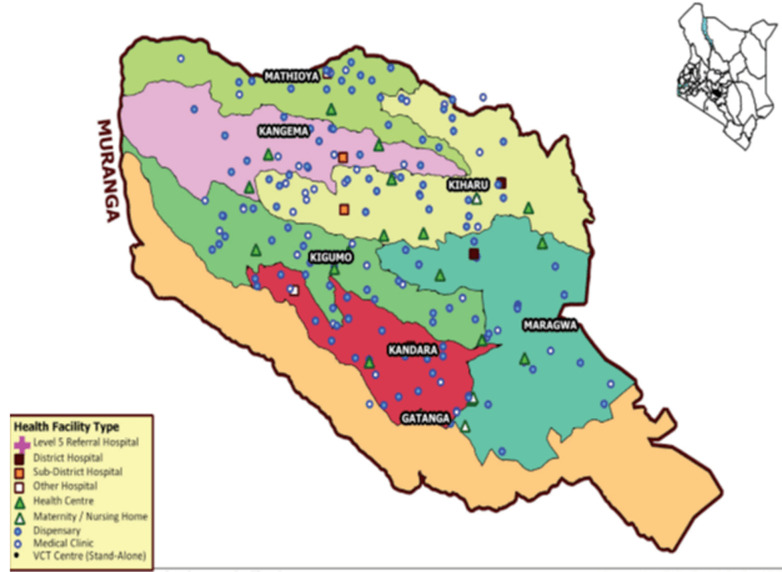
central region of the Republic of Kenya

**Study design and participants:** between July and December 2018, during a humanitarian medicine health care project in association with County health authorities from Murang'a (Kenya), we performed a joint intervention about the strategies for improving care management of hypertension and diabetic patients. This study was supported by the Department of Health from Murang'a County at Maragua Hospital. Murang´a County is one of the five counties in the Central region of the Republic of Kenya (Figure 1). The county occupies a total area of 2,558.8Km2 and has a population of 1,056,640. It has 7 public hospitals and 106 primary health care facilities. We visited 9 health facilities in Murang'a County from different health care levels, classified according Kenyan health system (Table 1).

**Table 1:** classification of hospitals in the Kenyan health system

Level	Resources
Level 1	Community services
Level 2	Dispensaries and clinics
Level 3	Health centers, maternity and nursing home
Level 4	Sub-county hospitals and medium-sized private hospitals
Level 5	County referral hospitals and large private hospitals
Level 6	National referral hospitals and large private teaching hospitals.

**Procedures:** first a NCD committee* consisted of 2 non-governmental international medical organizations (Cirugía Solidaria and, VIHDA Association), Murang'a County health authorities and members of the hospital management teams from the selected hospitals was formed. We developed a multidisciplinary NCD team** to carry out face-to-face in-depth retrospective interviews as part of a survey on management of Diabetes and Hypertension (Suppl. Info 1). The facilities included in the survey were hospitals (level 5, 4 and 3) with maternity services but with considerable busy outpatient services and offering diabetes and hypertension clinics.

### Data collection

This survey was based on the recommendations from WHO for these diseases (Suppl. Info 2) and we inquired about: a) health resources for the management of these diseases in the County of Murang'a, regarding hospital level, primary health care level and kind of health worker (specialist doctor, physician/ family doctor, clinical officer, nurse, community health worker and others); b) how these NCD are detected, how patients go to the specific diabetic and hypertension clinics, c) scientific knowledge of health care personnel about hypertension and diabetes, which therapeutic measures are carried out and how is follow-up and therapeutic measures are carried; d) existence of the basic and indispensable tools and essential medication that should be present in low resource clinics and that are recommended in the PEN document. We evaluated if they know and do the recommendations established in the PEN tools in their clinic. We focus on the kind of recommendations that should be made in primary and secondary prevention in hypertensive and diabetic patients with respect to both, the modification of lifestyles and the treatments that should be implemented and when. Specific questions were asked about whether all these strategies were being carried out. We also analysed the controls and actions recommended in this document to avoid complications in hypertensive and diabetic patients (measures to prevent chronic kidney disease, diabetic foot complications, diabetic retinopathy and neuropathy). We also request if there was a registry system in the clinic, we visited it and checked if there was any type of registry of diabetic and hypertensive patients who came to the hospital. They showed us the records, and we collected the data. We made a checklist where the professionals had to indicate which tools and/or drugs were available. The survey was carried out completely in the hospitals selected for the study. For this purpose, the NCD teams went to each facility and carried out an audit in which they interviewed the various professionals involved: doctors, nurses, health workers, recording managers, pharmacists and hospital management, and asked them about all the questions items contained in the survey (Suppl. Info 3). We also physically visited the facilities, the recording room, the clinics, and all the flow charts or protocols used for the management of these patients in the hospital. A summary report was compiled in order to assess the areas of improvement. The report was shared with the NCD team and the health authorities.

**Measurements and definitions:** for assessment of the prevalence in the populations of individual risk factors, hypertension was defined as a measured blood pressure ≥ 140 mmHg systolic and/or ≥ 90 mmHg diastolic. Treatment for hypertension was defined when indicate to take drug treatment for hypertension. Controlled hypertension was defined who have a blood pressure below 140 mmHg systolic and 90 mmHg diastolic. Diabetes mellitus was defined as the presence of non-fasting blood glucose of ≥ 11.1 mmol/L, or fasting blood glucose of ≥ 7.0 mmol/L, or self-reported use of drug treatment for diabetes. Cholesterol was classified based on the Adult Treatment Panel III classification, with high cholesterol defined as ≥ 6.2 mmol/L.

**Statistical analysis:** the survey made use of a semi-structured questionnaire, while the units of analysis for the survey were households. The questionnaire included details regarding hospital characteristics like setting type (rural, urban or semi-urban), type of health worker responsible for primary care, number of primary care clinics and protocols of diabetes and hypertension management. In order to ensure quality control, reliability and precision of data collected, the investigators went to the hospital and conducted in-depth interviews with health care workers completed questionnaires were checked on the spot and re-checked in the office for validation before data entry Qualitative variables are expressed as frequencies and percentage, quantitative variables are expressed as mean and standard deviation. Data was entered in MS-Excel and was analyzed using the Statistical Package for Social Sciences (SPSS) Window version 22.0.

## Results

A total of 9 health facilities were included in the study. We visited 1 Level 5 hospital (Murang'a County Referral Hospital), 3 level 4 hospitals (Maragua Sub county Hospital, Muriranjas Sub county Hospital, Kiria-ini Mission Hospital), 4 level 4 primary health care centers (Kangema Sub county Hospital, Kigumo Sub county Hospital, Kandara Sub county Hospital and Kirwara Sub county Hospital) and 1 level 3 primary health care center (Makuyu Health Center). Six hospital were rural, 2 semi-urban and 1 urban. The median subpopulation area and hospital area were 179869 (148289,75-202810,5) and 28086 (IQR, 24020-465000), respectively. All of the health facilities that we visited had specific diabetes and hypertension clinics, a health records officer and a patient´s registry (Table 2). The number of patients with diabetes and hypertension registered in public hospitals in Murang'a County were 6628 (0,45%) and y 6694 (0.45%), respectively. Six hospitals had combined hypertension and diabetes clinics and 3 had separated clinics for each disease.

**Table 2:** summary of the health facilities surveyed, health records officer and a patient´s registry

	1	2	3	4	5	6	7	8	9
HOSPITAL	Maragua Hospital	Muranga County Referral Hospital	Muriranjas Hospital	Kangema Subcounty Hospital	Kiriaini Mission Hospital	Kigumo Hospital	Kandara Hospital	Kiwara Subcounty Hospital	Makuyu Health Center
Setting type	Semi-urban	Urban	Rural	Rural	Semi urban	Rural	Rural	Rural	Rural
Type of worker: community health worker	0	10	0	60	0	0	0	0	0
Type of worker: nurses	1	1	1	1	0	1	1	3	0
Type of workers: general doctors	3	6 clinical officer and 3 resident	3	2 medical officer and 8 clinical officers	2	2 medical doctors	2	2	1 medical officer / 3 clinical officer
Type of worker: specialist doctors	0	1	0	0	1	0	0	0	1
Type workers: others	1 health record officer	1 nutricionist	1 nutricionist/1 record officer	Nutricionist 2	Nutricionist 1	Nutricionist 2	1 nutricionist/ 1 phisiotherapist	nutricionist	NA
PCL/Hospital	Hospital	Hospital	Hospital	PCL (level 4 but only with maternity	Hospital	PCL (level 3 hospital)	PCL (level 3)	Level 4	PCL
Number beds	131	250	127	NA	120	NA	NA	6	NA
Population	43000 hab	1200000	24258	23783	10000-80000	28086	34000	25249-195810	9200-182254
Number prymary care clinics in area	12 dispensaries 3 health care	NA	NA	3 health center and 14 dispensaries	NA	NA	5 health centers	Dispensaries 21 Health center 5	NA
Number visits/month	320 registry	253 registry	250registry	320	2490 (electronic register)	593	250	206	161
Number visits/ day	40 registry	50 registry	30/clinic	40 patients /each clinic	21-40/clinic	45-50 (electronically register)	25/ clinic	40-45	35-50
Number first visit Hypertension clinic/ January to august 2018	1061	333	NA	210	NA	265	363	217	18
Number first visit Diabetic clinic /January to august 2018	446	211	NA	63	NA	23	203	83	14
Number revisits Diabetic clinic / January to august 2018	NA	921	NA	1565(combined)	457 (mixed new and revisted)	150	437	NA	468
Number revisits Hypertension clinic/ January to august 2018	NA	948	NA	1565(combined)	1591(mixed new and revisted)	2356	597	NA	1149

**Patient records and Medical registers:** six hospitals used protocols for patient management and 3 used flow charts. Only in 3 clinics, the doctors knew about WHO/ISH risk predictors charts and used them. All hospitals had medical information registers and clinical patient records. Only one hospital (Kiriaini Mission Hospital) had electronic records. The Ministry of Health through the NCD department has standardized diabetes and hypertension patient files and registers (permanent and daily register). However, there was need for distribution of hard copies across all the county facilities.

**Clinics pathway and flow of patients:** we personally assessed in 3 hospitals the flow of diabetic and hypertensive patients. Patients were diagnosed in dispensaries, health centers, maternities, obstetric clinics, casualty or in another health facility. Some patients were diagnosed when they self-referred to the outpatient clinic for some complaints and the clinical officers conducted a blood pressure (BP) screening and glycaemia (only based in some clinical criteria). Afterwards, they were booked for diabetic/ hypertensive clinics. When the patients came with an appointment, first they went to the registry office, and then the nurse´s station for vitals assessment including BP, before going to the respective NCD clinic.

### Medical and clinical resources

**Figure 2:**
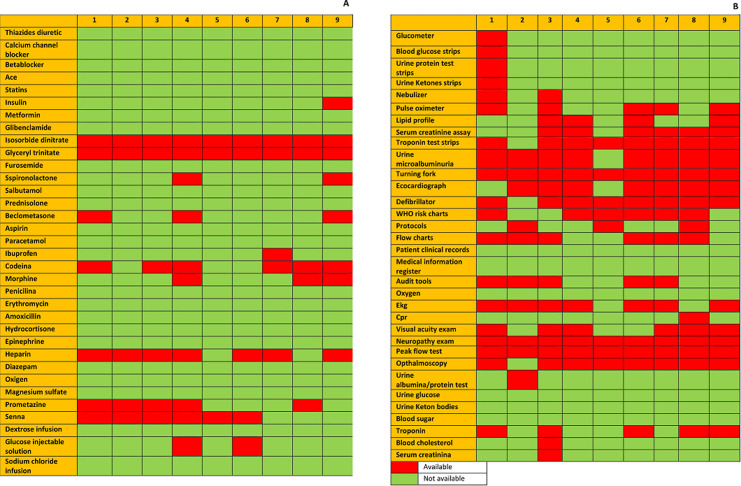
list of the availability of clinical and medical resources in the different hospitals surveyed in the management of diabetes and hypertension

We asked in the questionnaire about the essential technologies and tools to manage NCD in the clinic (Figure 2). All the hospitals had all the basic instruments (thermometer, stethoscope, glucometer, nebulizer and glucose, proteins and ketones urine strips test) but only 4 hospitals had pulse-oximeter. No hospital had a tuning fork, spacers for inhalers, peak flow meter, neither ophthalmoscope. Only one hospital had ECG, but they didn´t use because they didn´t have the necessary skills. Five hospitals had a blood test (serum creatinine, lipids profile, cholesterol). Only one hospital (Murang'a) had troponin test. All the hospital performed the basic procedures recommended (administer oxygen, injections, fluids administration, cardiopulmonary resuscitation, and ambulance referral). Only 3 of the clinics performed visual acuity examination and no facility did neurologic examination for neuropathy complications. The hospitals had most of the recommended essentials medicines (see survey) but no hospital stocked Isosorbide dinitrate and Glicerine trinitate. Only 3 hospital pharmacies had codeine and Senna, and only 4 had Promethazine in stock. All public hospitals offered subsidized medicines and consultations. In all the level 4 and 5 hospitals, patients copay for services or use National Health Insurance Fund. In the level 3 hospitals and below, all services are free of charge.

### Clinical management

We enquired during the survey if the health care workers perform the essential interventions for primary and secondary prevention as recommended by WHO in NCD (see annex I survey protocol). All the medical doctors interviewed knew these recommendations, and they applied most of them in their clinical practice. They only had issues with prevention of microangiopathic diabetic complications. Regarding optimal glycemic control to prevent complications, all the medical doctors knew that they had to do it and tried to do it. They didn´t conduct the HbA1 test (though it is recommended in the guidelines) because no public hospital had the test available. A 66% sent patients go to private laboratories and the remaining patients try to control with glucometer or blood glucose. A 33% of doctors recognized that they didn´t do a good control due to several reasons such as patients cannot pay HbA1 test, discontinuation of treatment because the hospital couldn´t give treatment for all period required (hospital pharmacist didn´t have enough stock).

A 4 of the hospitals did prevention of foot complication. Some of the measures included health talks by the nutritionist, and educational charts on the consultation room's wall. The rest of the health workers only examined the feet if patients had complaints. Some of the reasons cited by most healthcare workers for not doing preventive examination of the feet for complications were lack of enough human personnel, lack of tools for foot examination, lack of knowledge, training needs, too many patients in the clinic which impede conducting a complete examination, and consultation rooms not adequate for complete physical examination. None of the hospitals did microalbuminuria test to evaluate kidney function. But all of them used an angiotensin converting enzyme inhibitor, when the patient needed because of BP. To prevent retinopathy, only in 2 hospitals, health workers tried to do fundoscopy once a year for all patients on follow up. For the patients with visual problems and needed diagnosis or treatment they were referred to ophthalmologists based in other referral centers (Murang'a CRH, Kenneth Matiba hospital, Thika level 5 hospital, and Kenyatta National Hospital). To prevent neuropathy, most of the hospitals tried to do optimal glycemic control. However, they did not do complete neurological examination because they didn´t have the examination tools (only 2 doctors had a patella hammer and none had a tuning fork), All hospital had no communication problems with other health facilities. In case of an emergency, they would consult on the phone, and if necessary, they would refer the patients using standard patient referral forms. Ambulance services were available across the county hospitals. After conducting the surveys, the NCD team concluded that one of the major weakness was on management of vascular complications in Murang´a County. The overall strengths and weaknesses are summarized in Table 3.

**Table 3:** weak points/ strong points for NCD (hypertension and diabetes) in Murang’a health system

Strong points	Weak points
High implication of authorities in NCD	Increase hypertensive and diabetic patients diagnosis
High implication of hospitals doctors in NCD	Improving: pzérevention diabetic complications
Medical doctors had good level of knowledge about these diseases following guidelines in the clinical practice	Improving lab resources: Hb A1 and microalbuminuria
There are national guidelines updated and a comprehensive training curriculum with job aids	Improving diagnosis and treatment of isquemic heart diseases
New registers and forms are available for these deseases	Improving control of glicemia and blood preassure. Better adherence to medication

**Training in NCD diseases:** sixty-six (66%) of the doctors and nurses had been trained at least once on NCDs. Fifty-five (55%) of the other health care workers had been trained once too. The MOH through the NCD department has been conducting a comprehensive 3 days training on diabetes and hypertension management and use of the new tools and registers. This has been carried out in Murang´a as reported by the NCD coordinator.

## Discussion

The results of these surveys show that the recommendations of the guidelines are not achieved, not because health workers do not know the scientific evidence and what they should do, but because of a lack of medical and clinical resources and the delay of diabetic and hypertensive patients in being treated to the health system. Therefore, when patients are assisted in hospitals, they already present serious complications (micro and macro) that are stable and are difficult to resolve. With the results obtained from the survey, we carried out an analysis, trying to prioritize the areas which need improvement, following the recommendations established by the country's own national guidelines. In order to prevent diabetic complications (microangiopathy and macroangiopathy) an optimal glycemic control is mandatory. Guidelines recommend the use of HbA1 for optimal glycemic control, with normal levels lower than 7% [11-14]. All medical doctors we visited knew this issue, but it was not possible to do that test in most of the public hospitals in Murang'a County due to the lack of reagents. The only hospital that did the test most of the time was the county referral hospital. During the survey, the average price of the test was 1500 ksh ($1.27). Most patients had to get the test at private facilities, with the majority not able to afford it. This makes it difficult for the doctors to properly monitor the glycemic control of the patients. The only readily available tests used were random blood sugar and fasting blood sugar.

The survey found out that microalbuminuria test was not done in any of the hospitals, both private and public, in Murang´a County. Therefore, this test it is not being used to monitor kidney function in diabetic and hypertensive patients to prevent chronic kidney disease as per guidelines recommendations [11,15,16]. Levels of microalbuminuria are of great diagnostic relevance in diabetes for early diagnosis of diabetic nephropathy; and in patients with hypertension, as an indicator of end-organ damage associated with a lowered life expectancy. The hospitals however used proteinuria, which is not so useful as a marker for the prevention of kidney disease. Based on the data collected during the survey, we did an estimation of the number of hypertensive and diabetic patients who had been diagnosed in the hospitals of each area for the last one year. There were fewer patients diagnosed compared to the national projections expected per both diseases. We found an undiagnosed microvascular complication in diabetic and hypertensive patients. Even though health workers are aware of the complications of diabetes, most do not carry out the necessary examinations to detect them in their daily routine. They didn't have all the necessary tools and instruments for doing it. Despite the recommended protocols being available, hard print and soft, more training in the management of these complications would be needed.

As considerations on the results obtained in the present study, it will be necessary to increase the detection and diagnosis of hypertensive and diabetic patients in Murang´a County. To this end, treatment of hypertensive and diabetic patients at the primary care level should be recommended [7,17-19]. Blood pressure and glycaemia control could be done at dispensaries and health centers. These will probably increase the number of patients detected. The diabetic and hypertensive clinics at the level 4 and 5 hospitals would be only for complicated cases. In other African countries there are published initiatives to improve this [20] and the Kenyan government is aware of this situation and at present has plans to try to improve it [21-23]. It is important to integrate blood pressure and glycaemia tests for all patients (meeting criteria) in all outpatient clinics in all health facilities, in order to reduce the problem of undiagnosed patients. Many experts recommended emulating the HIV clinics model. Although HIV and TB are characterized as Communicable Disease, they are managed using a chronic care model. Patients follow up is regular by medical providers and adherence to long-term medication regimens. These decentralized models of healthcare is a key feature of effective chronic care delivery [24].

Another point of interest is the lack of management of acute coronary syndrome in the county hospitals: the absence of EKG machines, the lack of knowledge about its management and the lack of key drugs in pharmacies for its management are very worrying. There were no EKG machines in most of the hospitals that we visited. Only Maragua and Murang´a CRH had EKG devices, but they didn´t use them because they lacked the necessary training. No hospital had two of the WHO essentials recommended drugs which are used for ischemic cardiac pain (Isosorbide dinitrate and glyceryl trinitrate) [7]. That means that in Murang'a Hospitals there is no accurate diagnosis and treatment of acute ischemic heart disease, increasing the morbidity and mortality of patients. It is also likely that cardiac disease is underdiagnosed in the county. Not much is known about the incidence and management of myocardial infarction in sub-Saharan Africa countries [25]. Studies show that there is also not much knowledge in the population of African sub-Saharan countries about the symptoms of myocardial infarction and its prevention [26-28]. Ischemic heart disease is the leading cause of mortality worldwide, it is very important to implement strategies to improve the knowledge of the general population and of the health workers. It's likely that heart disease is killing many undiagnosed people in Africa.

The weakness of our research is that we were unable to obtain reliable records of hypertensive and diabetic patients from each facility. It is likely that a lot of data was missing from the records we consulted. On the other hand, our survey did not take into account the opinion of the patients, so we were not able to evaluate the degree of knowledge of the people in the county about these diseases. Obviously, a low knowledge of these diseases can also be influencing the patients' low utilization of the health system. Some patients didn´t attend clinics in their respective hospitals. It was suspected that some patients went to other hospitals outside the County (e.g. Thika level 5 Hospital or private facilities) therefore explaining the lower numbers found. Some people went to the dispensary or health care center and were diagnosed there, but didn´t attend NCD clinic in the hospitals. Some people are still undiagnosed. WHO has warned in several studies of the existence of undiagnosed people with NCDs in low and middle income countries due to both lack of awareness of the population and lack of screening of the health system [19-21]. In addition, there were substantial differences regarding the diagnosis of these diseases in each area. It would be necessary to have more information about the total health facilities in the county (primary health care and hospitals) to analyze these data and to better understand the unequal distribution of patients.

## Conclusion

In conclusion, we suggest that it is necessary to improve the diagnosis and treatment of patients with hypertension and diabetes in Murang'a County at the various levels of health care (from level 1 in the community to level 5 in the referral hospital). There is need for quality epidemiological data from both diseases to inform practice and policy.

### What is known about this topic

- It is a priority to know the situation regarding the real number of hypertensive and diabetic patients in each county is and how these diseases and their complications are being managed in Kenyan health system.

### What this study adds

- This study reveals that it is necessary to establish screening methods in order to detect all patients, start treatment earlier and follow up more closely on the possible complications of these diseases;
- For this, it is necessary to transfer the diagnosis and treatment of these patients at primary care level;
- We must continue working to improve these diseases in Kenya.
